# Improving primary care management of asthma: do we know what really works?

**DOI:** 10.1038/s41533-020-0184-0

**Published:** 2020-06-17

**Authors:** Monica J. Fletcher, Ioanna Tsiligianni, Janwillem W. H. Kocks, Andrew Cave, Chi Chunhua, Jaime Correia de Sousa, Miguel Román-Rodríguez, Mike Thomas, Peter Kardos, Carol Stonham, Ee Ming Khoo, David Leather, Thys van der Molen

**Affiliations:** 1 Asthma UK Centre for Applied Research, Usher Institute, University of Edinburgh, Old Medical School, Teviot Place, Edinburgh, EH8 9AG UK; 2 Department of Social Medicine, Faculty of Medicine, University of Crete, Heraklion, Greece; 3 General Practitioners Research Institute, 59713 GH Groningen, The Netherlands; 4 University of Groningen, University Medical Center Groningen, GRIAC Research Institute, Groningen, The Netherlands; 5 Observational and Pragmatic Research Institute, Singapore, Singapore; 6 Department of Family Medicine, 6-10 University Terrace, University of Alberta, Edmonton, AB T6G 2T4 Canada; 7 Peking University First Hospital, Beijing, China; 8 Life and Health Sciences Research Institute (ICVS), School of Medicine, University of Minho, Braga, Portugal; 9 33ICVS/3B’s, PT Government Associate Laboratory, Braga/Guimarães, Portugal; 10 Primary Care Respiratory Research Unit, Instituto de Investigación Sanitaria de las Islas Baleares (IdISBa), Palma, Spain; 11 Department of Primary Care, Population Sciences and Medical Education, University of Southampton, Southampton, SO16 5ST UK; 12 Respiratory, Allergy and Sleep Unit at Red Cross Maingau Hospital, Friedberger Anlage 31-32, 60316 Frankfurt, Germany; 13 NHS Gloucestershire Clinical Commissioning Group, Brockworth, UK; 14 Department of Primary Care Medicine, Faculty of Medicine, University of Malaya, 50603 Kuala Lumpur, Malaysia; 15 Global Respiratory Franchise, GlaxoSmithKline plc., GSK House, 980 Great West Rd, Brentford, Middlesex TW8 9GS UK; 16 Department of General Practice, University Medical Center Groningen, University of Groningen, Groningen, The Netherlands

**Keywords:** Asthma, Health policy

## Abstract

Asthma imposes a substantial burden on individuals and societies. Patients with asthma need high-quality primary care management; however, evidence suggests the quality of this care can be highly variable. Here we identify and report factors contributing to high-quality management. Twelve primary care global asthma experts, representing nine countries, identified key factors. A literature review (past 10 years) was performed to validate or refute the expert viewpoint. Key driving factors identified were: policy, clinical guidelines, rewards for performance, practice organisation and workforce. Further analysis established the relevant factor components. Review evidence supported the validity of each driver; however, impact on patient outcomes was uncertain. Single interventions (e.g. healthcare practitioner education) showed little effect; interventions driven by national policy (e.g. incentive schemes and teamworking) were more effective. The panel’s opinion, supported by literature review, concluded that multiple primary care interventions offer greater benefit than any single intervention in asthma management.

## Introduction

Asthma is a common chronic condition that is estimated to affect 339 million people worldwide^1,2^. Despite major advances in asthma treatment and the availability of both global^2^ and national guidance, asthma continues to cause a substantial burden in terms of both direct and indirect costs^1^. In 2016, estimated worldwide asthma deaths were 420,000^1^ and although there have been falls in some countries over the last decade, significant numbers of avoidable deaths still occur^3^. Mortality rates vary widely, with low- and middle-income countries faring worse^4^. For example, Uganda’s reported mortality rate is almost 50% higher^5^ than that reported globally (0.19/100,000)^6^, although inter-country comparisons using different data sources and epidemiological methodologies have limitations. The World Health Organisation (WHO) has a global ambition for universal healthcare coverage by 2030 as millions of people worldwide do not have accessible affordable medical care^7^. The WHO moreover recognises that health systems with strong primary care have the utmost potential to deliver improved health outcomes, greater efficiency and high-quality care^7^. Perversely the availability of good quality primary and social care tends to vary inversely, those having the greatest needs being least likely to receive it^8^.

In addition to the issues of access and the quality of care, both under- and over-diagnosis of asthma is common in all healthcare settings, but the issue is of particular concern in primary care, where most initial diagnoses are made^9,10^.

For people with asthma, high-quality, local and accessible primary care could be a solution to poor control^11^. Our aim was to identify the factors that experts believe enable the delivery of high-quality asthma care and to review the evidence that confirms that these factors do indeed have positive outcomes in primary care.

## Results

### Key drivers and their underpinning components

The expert panel identified five key drivers for the delivery of quality respiratory care in primary care and a number of components underpinning each of these drivers. These are summarised in Table 1.

**Table 1:** Key drivers and their underpinning components identified by the expert panel.

1. National healthcare policy
− Appropriately resourced primary care services
− Actions to support universal health coverage
− Recognition of importance of non-communicable chronic disease management
− Balance between public and private insurance: healthcare systems
− Redistribution of funding from hospitals to primary care
2. Clinical guidelines
− Recognition that primary care uses multiple disease guidelines
− Primary care ownership and succinct evidence-based guidelines
− Accessible guidelines produced in a standard recognised format
− Consider shifting to symptom-based guidelines
3. Reward for performance
− Recognition and rewards for high-quality respiratory practice
− Clearly defined financial incentive schemes
− Reward for the practice not individual practitioners
− Reimbursement policies aligned to guidelines, including prescribing
4. Practice resources and organisation
− Registered patient lists and fully integrated computer systems
− Clinical care pathways
− Access to high-quality lung function and other diagnostic tests
− Access literacy and culturally sensitive patient education
5. Workforce
− Specialist asthma training programmes in primary care
− Dedicated and appropriately asthma-trained personnel
− Collaborative working across the wider primary healthcare team, with defined roles
− Excellent interdisciplinary communication processes

Of the 50 articles selected from the review, there were comparatively smaller numbers of publications relating to the impact of National Health Policy and Guidelines. However, there was more substantial evidence relating to the other three key drivers, which is summarised in tabular format (Tables 2–4).

**Table 2:** Evidence summary to support reward for performance.

Reward for performance	Reference	Country	Study type	Description	Study outcomes
Clearly defined financial incentive schemes	Langdown and Peckham^17^	UK	Review of 11 studies	The UK quality and outcomes framework (QOF) one of the world’s largest pay-for-performance schemes.	The QOF has limited impact on improving health outcomes due to its focus on process-based indicators and the indicators’ ceiling thresholds.
Clearly defined financial incentive schemes	Gillam et al.^18^	UK	Systematic review of 94 studies	Quality of care for incentivised conditions during the first year of the QOF improved at a faster rate than the preintervention trend.	Modest improvements in the quality of care for chronic diseases.
Recognition and rewards for high-quality respiratory practice	Gillam et al.^18^	UK	Systematic review of 94 studies		Increased practice activity but limited evidence of improving the quality of primary healthcare or cost-effectiveness.
Reward for performance	Scott et al.^19^	Studies from the US and UK	Review of 7 studies	Pay-for-performance (P4p) schemes reviewed.	There is insufficient evidence to support or not support the use of financial incentives to improve the quality of primary healthcare
Reward for performance	To et al.^20^	Canada (Ontario Asthma Surveillance System	Three primary care incentive models evaluated	Quality measured using six validated, evidence-based asthma performance indicators (traditional fee-for-service model, the blended fee-for-service and blended capitation models).	Quality of asthma care improved over time within each of the primary care models. Blended fee-for-service and blended capitation models appear to provide better quality care compared to the traditional fee-for-service model.
Reward for the practice not individual practitioners	Kirschener et al.^21^	Netherlands	Observational study of 60 practices with a pre- and post-measurement	A P4p programme designed by target users containing indicators for chronic care, prevention, practice management and patient experience	After 1 year, significant improvement by +11.5% was shown for the process indicators for asthma.
Reimbursement policies aligned to guidelines, including prescribing	No studies found via search				

*P4p* pay for performance, *QOF* Quality and Outcomes Framework, *UK* United Kingdom, *US* United States.

**Table 3:** Evidence summary to support practice resources and organisation.

Practice resources and organisation	Country^(Reference)^	Study type	Description and study outcomes
Registered pt lists and fully integrated computer systems AND Clinical care pathways	UK^22^	Questionnaire; no data	SIMPLES, a structured PC approach to reviewing pts with uncontrolled asthma—encompassing pt education monitoring, lifestyle/pharmacological management and addressing support needs. Involves close cooperation between PC and SC. Outcomes: No data available.
Registered pt lists and fully integrated computer systems AND Clinical care pathways	NL^23^	Questionnaire; no data	SIMPLES adapted using a modified e-Delphi approach to assess the stakeholder opinion. Outcomes: Nine-component questionnaire—a robust and holistic approach for difficult-to-manage asthma. No data available.
Registered pt lists and fully integrated computer systems	UK^24^	Cluster-randomised trial in 29 PC practices with 911 at-risk asthma pts	Pilot study showed that PC intervention for targeted at-risk asthma patients had the potential for improving practice level management and reducing asthma emergency admissions.
Registered pt lists and fully integrated computer systems	UK^25^	Pragmatic, 2-arm, RCT; 270 PC practices covering >10,000 registered ‘at-risk asthma’ pts	Aimed to determine whether the creation and integration of at-risk asthma registers into PC reduces asthma-related crisis events for at-risk pts over a 12-month period compared to control practices. Outcomes: No data available.
Registered pt lists and fully integrated computer systems	UK^26^	Retrospective study; 26 at-risk asthma pts and 26 matched controls for 1 year pre- and post-intervention	Implementation/service use costs estimated before and 1 year after introduction of an at-risk register. More ‘at-risk’ than control pts were hospitalised/attended A&E/nebulised for asthma; also used out-of-hours services/attended GP/received OCS (all *p* < 0.025). Outcomes: After register introduction, no at-risk pts were admitted or attended A&E.
Registered pt lists and fully integrated computer systems	Multi-national (US, NL, AU, UK, DK)^27^	Systematic review of 19 studies representing 16 RCTs (2003–2013) evaluating CCDS for pts with asthma and COPD	Use of CCDS improved asthma and COPD care in 14 of the reviewed studies (74%). There was considerable improvement in healthcare process measures and clinical outcomes. The effect on workload, efficiency, safety, costs, provider and pt satisfaction remain understudied.
Registered pt lists and fully integrated computer systems	Multi-national (US, NL, UK, ES)^28^	Systematic review of 8 RCT CCDS (1990–2012) for professional asthma management	Use of CCDS by HCPs was found to be low, and adherence to the advice was limited. Concluded, if used, CDSS could result in closer adherence to guidelines and improve some clinical outcomes. Better alignment to clinical workflow would enhance their use.
Registered pt lists and fully integrated computer systems	NL^29^	1-year RCT; 200 adults (18–50 years) with mild–moderate persistent asthma	Pt groups: (i) weekly asthma control monitoring via online ACQ, treatment adjusted via self-management algorithm supervised by an asthma nurse specialist; (ii) usual care. Outcomes: Weekly self-monitoring/treatment adjustment led to improved asthma control in pts with partly/uncontrolled asthma at baseline.
Access to high-quality lung function testing and other diagnostic tests	Unknown at present^30^	Protocol: This will be a systematic review	Clinical prediction models can be used to aid PC asthma diagnosis by estimating outcome; models combine ≥2 predictors, e.g. clinical history/physical examination/test results/treatment response. Outcomes: No data available.
Access to high-quality lung function testing and other diagnostic tests	NL^31^	Observational study	An online support system to advise GPs on pt diagnosis and treatment. Spirometry performed by local GP laboratory; spirometry results, pt history questionnaire, ACQ and CCQ reviewed online by pulmonologist; who advises GP online, supported by a guideline-based algorithm. Outcomes: Number of pts with unstable asthma (ACQ ≥ 1.5) dropped from 245 to 137.
Access to high-quality lung function testing and other diagnostic tests	NL^32^	PC Diagnostic Centre study. 156 pts randomly selected from asthma/COPD-service referrals	Five respiratory specialists assessed spirometry data and pt histories. Facilities developed to provide spirometry testing by specially trained clinicians. GPs reluctant to perform or interpret spirometry themselves may be supported diagnostically by respiratory specialists in an asthma service although the reliability of this advice varies.
Access to high-quality lung function testing and other diagnostic tests	UK^33^	PC study; 678 pts aged 4–80 years with first FeNO assessment at index date	FeNO use to guide ICS initiation/dosing decisions and identify poor adherence. In the year following index date, FeNO use was evaluated in 2 pt cohorts to: (i) identify steroid-responsive disease; (ii) guide asthma management. Outcomes: Algorithms to guide practical FeNO use could improve diagnostic accuracy/asthma regimen tailoring.
Access literacy/culturally sensitive pt education		No studies found via search	

*ACQ* Asthma Control Questionnaire, *A&E* Accident and Emergency department, *AU* Australia, *CCQ* Common Cold Questionnaire, *CDDSS* Computerised Clinical Decision Systems, *COPD c*hronic pbstructive pulmonary disease, *DK* Denmark, *ES* Spain, *FeNO* Exhaled Nitric Oxide Test, *GP* General Practitioner, *HCP* healthcare practitioner, *ICS* inhaled corticosteroid, *NL* Netherlands, *PC* primary care, *PTS* patients, *RCT* randomised clinical trial, *SC* secondary care, *UK* United Kingdom, *US* United States.

**Table 4:** Evidence summary to support workforce issues.

Workforce	Country	Study type	Description and study outcomes
1, 2, 3	CA^34^	Retrospective database study, adults. 2008–2009. PC physician/network visited (*n* = 1,502,916); usual care (*n* = 1,109,941)	PC networks designed to facilitate access to interprofessional, team-based care, using AHPs skills in providing coordinated healthcare. Health outcomes associated with PC networks compared with conventional PC. Outcomes: Pts in network practices less likely to visit ED for conditions such as asthma; fewer ED visits and shorter hospital stays.
1, 2, 3	US^36^	Implementation study. 42 pharmacies, 2419 pts and 1284 provider interventions	Community pharmacist reviews of pts with poorly controlled asthma/no recent physician asthma review; physician referral was a service component. Outcomes: Benefits in asthma control, knowledge, inhaler technique, AAP ownership, ARQOL, and adherence.
1, 2, 3	AU^37^	A pragmatic cluster-randomised trial 96 pharmacists, 570 pts	Community-based asthma service by specially trained pharmacists: 3 vs. 4 visits in 6 months (12-month follow-up). Outcomes: Clinically important outcomes in both groups with minimal intervention, 3-visit service feasible/effective to implement, with 12-month review.
1, 2, 3	US^38^	Prospective pre-post study of pts receiving intervention for 9 months; 126 pts	Pts received physician−pharmacist collaborative management in PC. Pharmacists provided AAP/education/physician referral as necessary. Outcomes: Asthma-related ED visits decreased by 30% in the 9 months.
1, 2, 3	US^39^	5 community-based clinics Retrospective pre- and post-intervention analysis	A team-based education approach involving an electronic clinical quality management system; reminders/provision of AAPs by nurses. Outcomes: Increased AAPs prescribed, pt outcomes were not measured.
2, 3	UK^40^	Community-based, randomised, open-label pragmatic study	SLS; a collaboration between physicians, nurses, hospital staff and pharmacists linked using electronic pt health record, improving HCP communication. Outcomes: Improved asthma control (ACT increase).
2, 3	BR^44^	Implementation study 132 PC physicians & nurses Aim to decrease number of respiratory-related (Asthma/COPD) referrals	Educational intervention (matrix support, evaluated in PC): physicians/nurse training/support from specialists (e.g. tailored education/joint consultations/case discussions). Outcomes: referrals decreased by >50% from 13.4 to 5.4 cases/month (*P* = 0.09). An effective tool to improve asthma knowledge and promote changed PC/SC relations. Pt outcomes not measured.
4	US^41^	Implementation study. 57 practices, 15,508 pts Pre-post	CATP; a provider-level intervention to improve guideline use and asthma care (education and pt resources). Outcomes: CATP improved guideline care processes but not pt outcomes, of practices: 40.4% increased ICS use, 53.2% increased AAP use; 78.7% initiated/increased spirometry use.
4	US^42^	Implementation study (asthma pts 5–64 years) 12 months pre- and post-CATP implementation 9 practices; 2678 pts	Compared 12 months pre- and post-use of the CATP in PC practice. Outcomes: An improvement in asthma quality processes—increase in rate of asthma severity measurement and medication management, no change in outcomes across multiple domains: exacerbations, utilisation, symptom scores, and pulmonary physiology measures.
4	CA^43^	Pragmatic improvement study. 23 physicians, 25 AHPs; 12-month pre/post-intervention knowledge	Mentorship-based intervention with interactive education/hands-on training/ unstructured peer mentoring. Aimed to address PC underuse/quality of spirometry. Outcomes: Improved spirometry test acceptability, poor overall spirometry usage (remained < 40%), health outcome effects not measured.
5	DK^35^	Consultation guide based on GINA guidelines	Consultation included symptom evaluation, treatment, compliance, lung function, scheduled follow-up appointment based on asthma control level. Outcomes: Asthma control improved when a systematic asthma management approach was introduced/applied by dedicated nurses.
5	ES^45^	Cluster controlled implementation study 57 practices 400 PC physicians and nurses, 6/12 pre−post-intervention 7 control	GP practices received an education programme for use of respiratory health status tools. Outcomes: In intervention practices slight improvement in pts with a record of a health status score (ACT, CAT and/or mMRC), but absolute % score recorded was still relatively low (1.70%), even after intervention. No differences in clinical outcomes.
5	AU^46^	RCT aged ≥55 with asthma *N* = 58 intervention group *n* = 56 control group	Groups: brochure only (controls); person-centred education (intervention). Outcomes: Intervention pts had improved asthma control, adherence, AAP ownership, ARQOL and exacerbations over 12 months vs. control pts.
5	Global^47^	Literature review of 24 studies	Reviewing conceptualisation/practice in PC. Enablement influenced by: open communication style/longer consultations/pt centredness of HCP. Outcomes: 2 RCTs suggest enablement linked to better pt outcomes.
5	DE^85^	5-year programme 2006–2010. *N* = 109,042 in year 5	German asthma management programme. Outcomes: Enhanced care quality; improved symptoms/adherence/pharmacotherapy/hospitalisation.

1 = Dedicated and appropriately asthma-trained personnel; 2 = Collaborative working across the wider Primary HealthCare Team, with defined roles; 3 = Excellent interdisciplinary communication processes; 4 = Specialist asthma training programmes in PC; 5 = Dedicated and appropriately asthma-trained personnel.

*AAP* asthma action plan, *ACT* Asthma Control Test, *A&E* Accident and Emergency department, *AHP* Allied Health Practitioner, *ARQOL* asthma-related quality of life, *AU* Australia, *BR* Brazil, *CA* Canada, *CAT* COPD Assessment Test, *CATP* Colorado Asthma Toolkit Programme, *COPD* chronic obstructive pulmonary disease, *DE* Germany, *DK* Denmark, *ED* Emergency department, *ES* Spain, *GINA* Global Initiative for Asthma, *GP* General Practitioner, *HCP* healthcare practitioner, *mMRC* Modified Medical Research Council, *PC* primary care, *PTS* patients, *RCT* randomised clinical trial, *SC* secondary care, *SLS* Salford Lung Study, *UK* United Kingdom, *US* United States.

### National Health Policy

The expert panel reached an agreement that the political will to prioritise asthma and to support both primary care and respiratory disease were fundamental elements for the achievement of a sustainable change. In their opinion this required national and local programmes supporting the improvements. There was however little evidence published to support this opinion with respect to patient outcome as it is not the area of research that is commonly undertaken. A review of seven national European asthma programmes to support strategies to reduce asthma mortality and morbidity concluded that national/regional asthma programmes are more effective than conventional treatment guidelines^12^. One of the most well-known and successful national programmes in Europe, which has resulted in reduced morbidity and mortality and decreased costs, is the Finnish National Asthma Programme^13^. Programmes outside of Europe have also demonstrated the impact that prioritisation of primary care can have on respiratory outcomes. Changing structures and policies in South Africa and in Brazil may start to impact on primary care^13,14^.

### Guidelines

Few studies have explored the extent of adherence to guidelines for asthma management based on data provided directly by GPs. One study aimed to evaluate adherence to GINA guidelines and its relationship with disease control in real life. According to GINA guideline asthma classification, the results indicated overtreatment of intermittent and mild persistent asthma, as well as a general poor adherence to GINA treatment recommendations, despite its confirmed role in achieving a good asthma control^15^. In the US, nationally representative data showed that agreement with and adherence to asthma guidelines was higher for specialists than for primary care clinicians, but was low in both groups for several key recommendations^16^.

### Reward for performance

Pay-for-performance (P4p) schemes are those that remunerate physicians for achieving pre-defined clinical targets and quality measures—so based on value—that contrasts to schemes that are simply a fee-for-service payment, which pay for volume of activity (Data from Review Table 2). In the UK, primary care has moved towards group practices with P4p compensation in which performance is measured using several defined quality indicators^17,18^. A systematic review of 94 studies showed increased practice activity but only limited evidence of improvements in the quality of primary care or cost-effectiveness, despite modest reductions in mortality and hospital admissions in some domains^18^. In another review of seven studies from the US and UK, the effects of financial incentive schemes were found to improve patient’s well-being, whilst the effects on the quality of primary healthcare were found to be modest and variable^19^.

An evaluation of three primary care incentive models, namely a traditional fee-for-service model, a blended fee-for-service model and a blended capitation model, demonstrated that the quality of asthma care improved over time within each of the primary care models^20^. The model that combined blended fee-for-service with capitation appears to provide better quality care compared to the traditional fee-for-service model in terms of outcome indicators such as a lower rate of emergency department visits.

A P4p programme in the Netherlands containing indicators for chronic care, prevention, practice management and patient experience was designed by target users^21^. A study of 65 practices that implemented the programme showed a significant improvement in the mean asthma score after 1 year. It showed that a bottom-up developed P4p programme might lead to improvements in both clinical care and patient experience.

### Practice resources and organisation

Optimal patient care requires targeted and tailored management (Data from Review Table 3). The experts felt that the organisation of both the GP practice and the local healthcare system had an influence on the provision of high-quality care. Registered patient lists and fully integrated computer systems were its foundation. An approach called SIMPLES—developed in the UK, incorporated into a desktop reference tool by the International Primary Care Respiratory Group and adapted for use in the Netherlands^22,23^—identifies patients who have uncontrolled symptoms or difficult-to-manage disease and addresses preventable or treatable factors to guide their management. Electronic alerts in patient records have also been used to identify those at increased risk of an exacerbation, in order to modify care and treatment^24–26^.

A systematic review of the effectiveness of computerised clinical decision systems (CCDS) in the care of patients with asthma demonstrated improvements in healthcare process measures and patient outcomes^27^. Conversely another systematic review focussing on their implementation in practice concluded that the limiting factors were the lack of their regular use by healthcare practitioners (HCPs) and adherence to the advice offered^28^. These reviews both concluded that CCDS have the potential to improve patient outcomes, practice efficiency and produce cost-saving benefits if implemented^27,28^.

Computerised systems linked with internet programmes to monitor asthma control can also afford benefits for patients. One study identified that the use of both weekly internet-based self-monitoring using the Asthma Control Questionnaire (ACQ) and treatment adjustment using an online management tool resulted in significant improvements in ACQ^29^.

Clinical prediction models could theoretically aid the diagnosis of asthma in primary care but supportive evidence is currently lacking^30^. However, there is strong evidence that service models aimed at supporting primary care practitioners with the diagnosis or ongoing monitoring of patients result in improved accuracy and patient outcomes^31–33^.

### Workforce

The expert panel felt that having access to dedicated and appropriately trained personnel preferably as part of multidisciplinary teams was essential (Data from Review Table 4). This need was accentuated because of increasing GP workloads and a shortage of primary care physicians in many countries.

There was extensive evidence^34–40^ that a variety of models involving a range of healthcare practitioners within both the core primary healthcare team and extended community teams improve patient outcomes and healthcare process measures—such as an increased use of asthma action plans, improved medication adherence^36,39^—and reduces the use of emergency care^34,38^.

One approach in Canada is based on using primary care networks, in which additional non-physician healthcare providers are funded to help provide coordinated healthcare^34^. In these networks patients were shown to be less likely to visit the ED than patients in practices that were not part of the network.

Evidence from a range of countries supports the beneficial role of pharmacists, working alone or in teams^36–38^. In a study utilising community pharmacists to review patients with either poorly controlled asthma or no recent asthma review, there were benefits in terms of asthma control, inhaler technique, action plan ownership, asthma-related QOL and medication adherence^36^. The pharmacists were able to recruit patients and incorporate this as part of daily practice. Availability of referral to a physician was an important component of the service.

Evidence also indicates that education delivered by a variety of methods enhances the quality of care delivered and improves patient outcomes^41–45^. Approaches integrating education with other interventions, such as the Colorado Asthma Toolkit Programme (CATP) that combines education with decision support tools, electronic patient records and other online support materials, have been shown to have positive outcomes^41,42^. Another team-based approach that combined an educational intervention with the integration of an electronic clinical quality management system with a reminder system found that the number of action plans increased significantly^39^.

Patient education is an important factor for the improvement of self-management and asthma control. An educational programme from Australia demonstrated that patients who received person-centred education had improved asthma outcomes compared to those receiving a brochure only^46^. One review paper^47^ about patient enablement concluded that HCPs need to develop their understanding of this concept to integrate this into practice as the level of this is linked to better patient outcomes.

## Discussion

Primary care is pivotal to any health system; however, there is no universal definition of what we mean by primary care and certainly not one standardised model of care. Without focussing on a single model, we have attempted to bring together expert opinion and the most recent evidence on strategies that improve outcomes in asthma patients in primary care. To our knowledge the methodology used in this project has not been used before. The panel of experts who identified the key drivers were knowledgeable of asthma in primary care at a national level in their respective countries and globally. A literature search to investigate the individual key drivers and their underpinning components was undertaken using a keyword search. This identified many publications but very few measured the effect on patient outcome and those that did reported conflicting results. Furthermore, we found a paucity of research relating to the components relating to national healthcare policy and guidelines.

The evidence suggests that health systems that have primary care as a cornerstone and place asthma as a healthcare priority improve asthma care and improve outcome on patient level. The highly regarded Finnish asthma initiative carried out more than 25 years ago not only identified asthma as a national priority, but also placed primary care at the centre of the programme, recognising the key role of General Practitioners and nurses and greatly reduced asthma mortality and morbidity^48^. After the successful implementation of the Finnish asthma plan, many other countries and regions have attempted to implement similar initiatives^13,14^. For example, in Poland and Brazil, asthma burden was reduced utilising such a strategy^49^.

Poor health outcomes in asthma patients have been attributed in primary care to gaps between evidence-based recommendations and practice^50,51^. Studies show that adherence to clinical guidelines is poor, whatever the clinical setting, with the main barriers being time pressures and limited resources^52^, reflecting that it is not the guidelines per se that improve care, but it is the implementation of the recommendations.

Most guidelines are complex, lengthy and generally biased towards a secondary care perspective. The Global Initiative for Asthma (GINA) committee acknowledges the difficulty of implementing their recommendations in primary care, but they are almost exclusively developed by tertiary care physicians^2^. In the Netherlands, the Dutch Royal Society of General Practitioners writes its own guidelines, which are all presented in the same recognisable brief format. Their asthma guidelines were first published in 1986 with revisions every 4 years and are relatively well followed^53^. However, there are now 194 different clinical guidelines in the Netherlands, illustrating just how difficult it is for General Practitioners to adopt all the recommendations of each clinical guideline and its update.

A survival analysis of guidelines has concluded that 86% are still up to date 3 years after their publication and yet the median lifespan of a clinical guideline is about 60 months^54^. New evidence is continually emerging and this implies that regular updates of clinical guidelines are necessary^55,56^. It is therefore important that all guidelines have a process for regular scrutiny^57^ and are updated for contemporary applicability. Indeed, asthma and COPD guidelines published by the Association of Scientific Medical Societies in Germany and the Asthma Guidelines of the German Respiratory Society are regularly updated, at least every 5 years (more frequently as necessary); if not they are deleted from the website.

The proliferation of guidelines and their asynchronicity can result in conflicting recommendations. For example, in the UK, four asthma guidelines could be followed (the GINA Report, British Thoracic Society and Scottish Intercollegiate Guidelines (BTS) and the NICE recommendations next to local guidelines)^2,58,59^, none of which are fully aligned. A review of three contemporaneous international guidelines updated in 2012 (The Canadian Thoracic Society (CTS), BTS and GINA) also revealed significant inconsistency arising from varying approaches to evidence interpretation and recommendation formulation^60^.

Globally, there is a move away from pure fee-for-service payments towards primary care payment schemes linked to performance, which recognise and reward good practice to improve quality and reduce costs^61^. These schemes combine quality standards and targets but still tend to be process driven, not outcome based. The evidence for the effectiveness of such schemes in general on improving quality of care is both inconclusive and inconsistent^62^.

The UK quality and outcomes framework (QOF), which includes asthma, is the world’s largest primary care payment for performance (P4p) scheme^63^. Evidence however shows that improved patient outcomes may not be sustained, cost reduction is unproven^18^ and leads to increased GP activity, but this does not necessarily correlate with improved individual patient benefit^64,65^. Furthermore, in Portugal, the recording of asthma and COPD prevalence as performance indicators in pay-for-performance contracts showed a modest but steady increase over time in physician’s diagnosis and ICPC-2 coding of these two conditions, but no direct patient benefits^66^.

Disease-specific schemes are usually aligned to clinical guidelines and some focus on prescribing. In Norway, under such a scheme, combination asthma medications were only reimbursed for patients diagnosed with asthma. As a result, asthma diagnosis significantly increased^67^.

The effect on health inequalities has also been studied. The results from UK QOF have shown that the gap between achievements from practices in the most deprived and least deprived areas narrowed^68^. Nevertheless, inequalities in morbidity and premature mortality persisted^69,70^. Additionally incentives can increase inequalities because those conditions that are ‘incentivised’ are afforded greater priority and resource allocation, to the detriment of those that are not^71^.

It would appear that simplistic fee-for-service schemes based purely on an activity—such as performing spirometry tests—which are not part of reimbursement of a more comprehensive assessment, have the potential to inadvertently lead to an increase in unnecessary tests. Pay-for-performance schemes have the potential to improve asthma care, but will be reliant on the specifics of the scheme and the quality indicators applied. They can be useful as part of a wider programme to raise quality and afford benefits over rewarding fee-for-service activity.

Appropriate practice organisation and systems focussing on the identification, diagnosis and treatment are pivotal for quality asthma care. There was compelling evidence to indicate that integrated, multi-faceted practice-based approaches for the management of patients improves outcomes and reduces the need for referral to secondary care^22,25,72^. Coordinated practice systems that combine several interventions such as decision support tools, flagging of electronic records, use of care pathways, staff training and structured approaches to patient education, if consistently implemented, afford the greatest benefits. Implementation of practice schemes is likely to be enhanced where there is dedicated clinical and administrative leadership.

Intuitively an accurate diagnosis should lead to better patient outcomes, although we found conflicting evidence that access to proper diagnosis has an impact on patient outcomes^33,73^. Nevertheless, an accurate diagnosis remains the fulcrum on which optimal asthma management depends. Indeed programmes in which an expanded medical team improved the quality of asthma care within the primary care setting (such as a diagnostic and management support organisation) show clear benefit on patient outcome^32^.

Spirometry combined with an assessment of reversibility has been set as gold standard for asthma diagnosis^2^. However, standards on quality of spirometry such as those set by the ERS and ATS are often not achieved^74–76^ and impose an unnecessarily high and potentially unachievable threshold in primary care^73^. Nevertheless, some studies have demonstrated that primary care office spirometry can meet the acceptability criteria^77–79^. Although such standards are laudable particularly in a specialist setting, their practicability in primary care, where patients commonly have mild–moderate, intermittent disease, is debatable. The latest ATS-ERS spirometry guidelines (published in October 2019) may address some of these issues.^80^ However, the use of spirometry in the diagnosis of asthma remains beyond reach in primary care around the world.

In many countries primary care physicians have limited or no access to tests of lung function or airway inflammation. The creation of diagnostic hubs in the community may open access to these tests^32^. A structured approach to diagnosis including applicability and feasibility for primary care is currently under development by an ERS taskforce; its outcome not available at the time of writing.

With rising clinical workloads, increasing clinical complexity and in many countries a shortage of trained primary care physicians, multi-professional teamworking is increasingly important.^81,82^ This is accentuated by the expectation for primary care to manage patients with chronic illness.

In many parts of the world, appropriately asthma-trained personnel, such as primary care nurses, are key to the delivery of high-quality asthma care. Dedicated nursing staff can offer continuity to patients, providing education and routine follow-up^35^. Evidence supports the concept that pharmacists working alone or in teams in collaboration with GPs are an accessible asset for the effective management of asthma and can positively influence asthma outcomes^36^.

Healthcare practitioner education is pivotal and the need for guideline-focused training in primary care is well established^82^. The literature seems to support this viewpoint but in many studies the effect on outcome has not been adequately considered, highlighting a need for more outcome-focussed research. Healthcare systems faced with the challenge of moving the care of people with long-term conditions such as asthma from established specialist services to primary care should consider implementing collaborative educational strategies^44^. Matrix-support collaborative care that includes training and support for primary care physicians/nurses from specialists, including joint consultations, case discussions and tailored education, has been shown to be well-accepted by primary care professionals and was associated with improved knowledge and reduced respiratory secondary care referrals^44^. A scoping exercise and literature review of the effectiveness of educational interventions in either changing health professional practice or in improving health outcomes was commissioned by The International Primary Care Respiratory Group (IPCRG)^83^. The impact of education interventions on their own was inconclusive, although there was some evidence of effectiveness when they are combined with other quality improvement strategies or incentives^83^.

Asthma continues to be a substantial cause of morbidity and mortality worldwide and there is need for a coordinated effort to improve care. A well-resourced primary care service is central to the provision of accessible and effective asthma care. An expert team identified the drivers that could enable improvements in both clinical management and patient outcomes, and a literature search showed that each of these individual drivers is supported by varying degrees of evidence. Objectively assessing the outcomes of such interventions is challenging because studies in this area are inherently complex, difficult to undertake and resource intensive, and so definitive research is seldom undertaken. In contrast single interventions studies are easier to conduct but frequently methodologically less robust and therefore tend to be inconclusive. Nevertheless, if substantial improvements in the management of asthma in primary care at a global level are to be achieved, combinations of interventions appear to be most effective. Well-supported holistic interventions involving the entire healthcare system and including the patient voice appear to provide the best outcomes.

## Methods

### Expert panel

An expert panel of 12 primary care global asthma experts—ten General Practitioners and two specialist nurses—was convened in Amsterdam. An initial teleconference between the panel preceded the meeting to gather ideas. The expert panel undertook a brainstorming exercise as part of a force-field analysis in order to reveal their ideas and experience regarding drivers of successful management of asthma in primary care^84^. A force-field analysis can be used to determine the forces (factors) that may prevent change from occurring and to identify those that cultivate change. During the brainstorming session, the experts were divided into facilitated groups to discuss the relative importance of the drivers and identify the factors which underpin each of them. Results were analysed thematically and circulated after the meeting for comment and agreement.

### Literature review

**Fig. 1:**
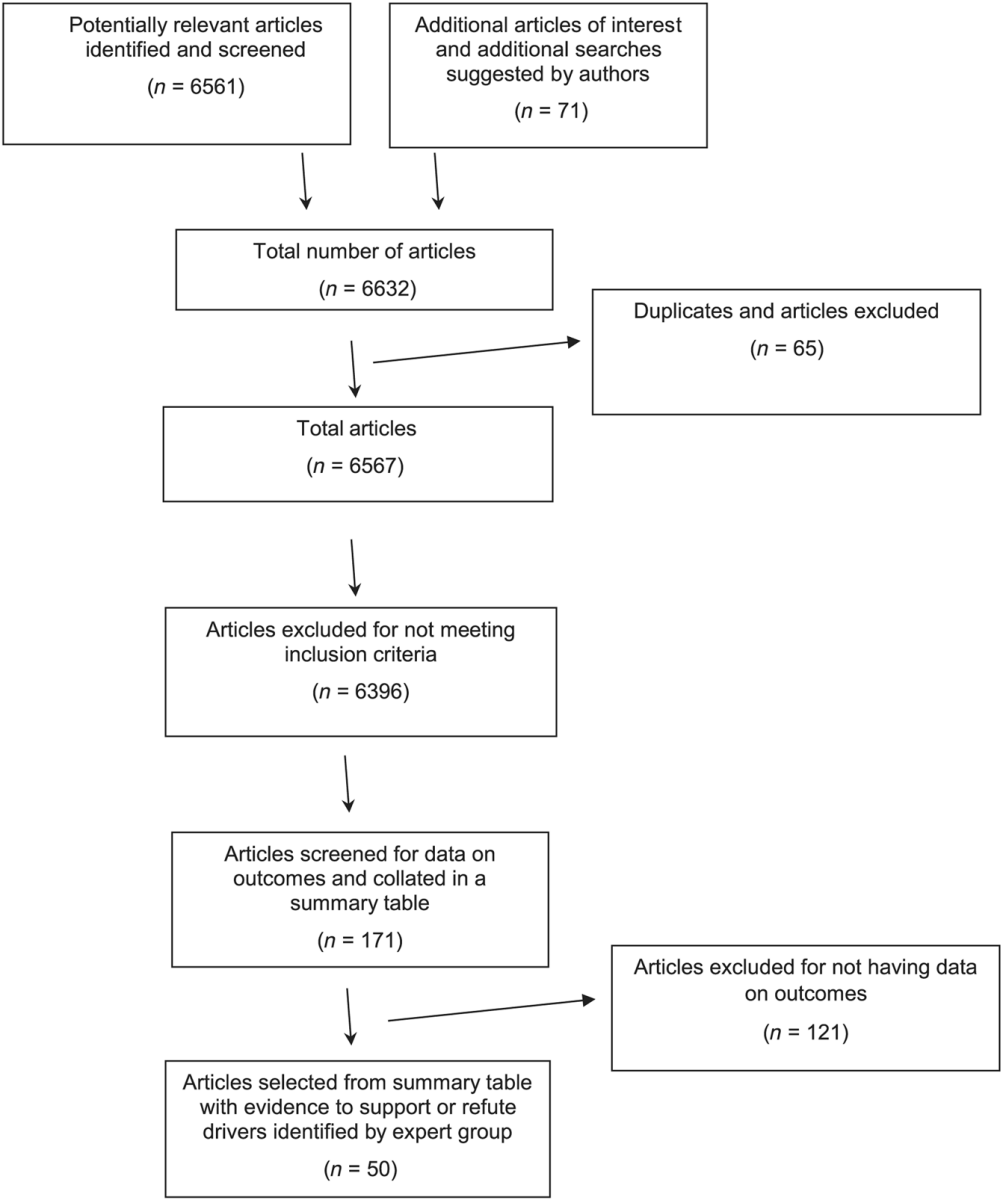
Flow of articles identified by literature review in PubMed. Process by which papers identified by literature review were subsequently screened for eligibility and the different stages in this process. This highlights the number of articles that were selected at each stage of the process, as well as the number of articles excluded and the reasons for exclusion. *n* number of articles.

To identify whether evidence existed for the drivers and factors identified by the expert panel, literature was searched from PUBMED using the terms asthma and primary care in combination with other terms listed in Table 5. Proposed search terms were combined using Boolean operators. The initial search was limited to papers published in English over the last 10 years and studies in adults aged over 18 years old. The experts were also asked for additional papers and in addition, more articles were identified from the references from the selected papers. Papers identified were subsequently screened for eligibility by MF and TM (Fig. 1). A total of 171 were included in the summary table of which 50 papers were identified as having evidence for the factors identified by the panel.

**Table 5:** Combinations of keywords used in PubMed search.

Asthma AND primary care; *n* = 6535
Asthma and primary care AND outcomes; *n* = 1502
Management of asthma in primary care AND outcomes, *n* = 821
Asthma AND primary care AND outcome AND improvement; *n* = 1728
Asthma AND primary care AND team building; *n* = 14
Asthma AND primary care AND team; *n* = 274
Asthma AND primary care AND incentives; *n* = 105
Asthma AND family practice AND outcome AND improvement in adults; *n* = 28
Asthma AND general practice AND outcome AND improvement in adults; *n* = 62
Asthma AND family practice AND adults; *n* = 950
Asthma AND general practice; *n* = 622
Asthma AND quality improvement; *n* = 455

## Data Availability

Anonymised individual participant data from this study and its associated documents can be requested for further research from www.clinicalstudydatarequest.com.
